# Finding the active genes in deep RNA-seq gene expression studies

**DOI:** 10.1186/1471-2164-14-778

**Published:** 2013-11-11

**Authors:** Traver Hart, H Kiyomi Komori, Sarah LaMere, Katie Podshivalova, Daniel R Salomon

**Affiliations:** 1 Donnelly Centre, Banting & Best Department of Medical Research, University of Toronto, Toronto, Canada; 2 Department of Molecular and Experimental Medicine, The Scripps Research Institute, La Jolla, CA, USA

## Abstract

**Background:**

Early application of second-generation sequencing technologies to transcript quantitation (RNA-seq) has hinted at a vast mammalian transcriptome, including transcripts from nearly all known genes, which might be fully measured only by ultradeep sequencing. Subsequent studies suggested that low-abundance transcripts might be the result of technical or biological noise rather than active transcripts; moreover, most RNA-seq experiments did not provide enough read depth to generate high-confidence estimates of gene expression for low-abundance transcripts. As a result, the community adopted several heuristics for RNA-seq analysis, most notably an arbitrary expression threshold of 0.3 - 1 FPKM for downstream analysis. However, advances in RNA-seq library preparation, sequencing technology, and informatic analysis have addressed many of the systemic sources of uncertainty and undermined the assumptions that drove the adoption of these heuristics. We provide an updated view of the accuracy and efficiency of RNA-seq experiments, using genomic data from large-scale studies like the ENCODE project to provide orthogonal information against which to validate our conclusions.

**Results:**

We show that a human cell’s transcriptome can be divided into active genes carrying out the work of the cell and other genes that are likely the by-products of biological or experimental noise. We use ENCODE data on chromatin state to show that ultralow-expression genes are predominantly associated with repressed chromatin; we provide a novel normalization metric, zFPKM, that identifies the threshold between active and background gene expression; and we show that this threshold is robust to experimental and analytical variations.

**Conclusions:**

The zFPKM normalization method accurately separates the biologically relevant genes in a cell, which are associated with active promoters, from the ultralow-expression noisy genes that have repressed promoters. A read depth of twenty to thirty million mapped reads allows high-confidence quantitation of genes expressed at this threshold, providing important guidance for the design of RNA-seq studies of gene expression. Moreover, we offer an example for using extensive ENCODE chromatin state information to validate RNA-seq analysis pipelines.

## Background

Second-generation sequencing technology has provided deep insight into the complexity of the transcriptome. Early sequencing of cellular mRNA resulted in a level of transcript quantitation that was in broad concordance with microarrays [1]. Subsequent studies with improved mapping tools [2,3] and increasingly deep sequencing depth [4,5] suggested that, with enough depth of coverage, most annotated genes could be observed at some level. A key unanswered question, however, is whether these low-abundance transcripts are biologically significant [6,7].

A recent study by Hebenstreit *et al*. [8] demonstrated that gene expression in mammalian cells measured by RNA-seq follows a bimodal distribution of high and low expression genes, and suggested that the high-expression genes comprise the active, functional transcriptome of the cell. The results of several studies constrain the range of the threshold that divides active from low-expression genes: at the upper bound, Hebenstreit *et al*. and Mortazavi *et al*. [9] calculated that fragments per kilobase of gene model per million mapped reads (FPKM) values of 1 to 2 correspond to ~1 mRNA molecule per cell, though a deep proteomic sampling of HeLa cells detected proteins from several genes expressed below this level [10]. At FPKM of about 0.3, RNA-seq reads were shown to map to exonic regions and intergenic regions at similar rates [11], suggesting lower confidence in measured expression below this level. However, the data used in these studies were from short read RNA-seq (often 32-base single-end reads) of moderate depth (typically ~20 million reads). Advances in RNA-seq library preparation and sequencing technology now regularly yield tens to hundreds of millions of paired-end reads of 50 to 100 or more bases in length. Increased read length improves mapping accuracy and lowers the odds of spurious multiple mapping, while greater read depth allows more accurate assessment of the relative abundance of low-expression transcripts as well as the detection (by at least one read mapping) of a greater number of genes [5]. These advances undermine the assumptions upon which previous heuristics for evaluating gene expression were based, highlighting the need for updated understanding of the signal and noise present in RNA-seq data.

In this study, we integrate current-generation RNA-seq and chromatin state data from the ENCODE project to understand the relationship between gene expression level and promoter activity signatures. We explore the effect of varying read depth on transcript detection and quantitation, and offer a novel normalization method that robustly identifies the subset of active genes observed in an RNA-seq experiment and provides guidance regarding efficient experimental design.

## Results and discussion

### Expression state from chromatin state

**Figure 1:**
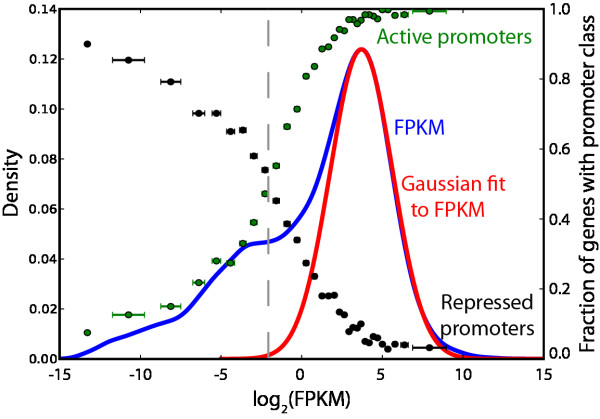
**A typical RNA-seq expression profile.** Blue, density plot of log-transformed FPKM values of protein coding genes, from ENCODE cell line GM12878 (left axis). Red, Gaussian fit to the right side of the main peak. Green, fraction of genes in bin (bin size = 500 genes) with an active promoter, from [14] (right axis). Black, fraction of genes in bin with a repressed promoter. The dashed grey line represents the level of gene expression where the fraction of active promoters is equal to the fraction of repressed promoters, and is evaluated in FPKM and as a Z-score relative to the Gaussian fit (zFPKM).

We examined the transcript levels of 17 human cell lines from the ENCODE 2.0 RNA-seq data set. Using the Tophat/Cufflinks pipeline [3,12], we determined gene expression levels of ~19,000 protein coding genes, using GENCODE gene models [13] (Additional file 1: Table S1). In all cases, and consistent with prior studies, the log_2_(FPKM) distribution shows a primary peak of high expression genes, with a long left shoulder of low-expression transcripts (Figure 1 and Additional file 2: Figure S1).

An important question arising from this observation is whether the low-expression transcripts of the shoulder are comprised of functional genes or merely by-products of leaky gene expression, sequencing errors, and/or off-target read mapping. To explore this question, we compared gene expression profiles to the results of an integrated analysis of chromatin state derived from ENCODE ChIP-seq data [14]. Each gene promoter was tagged as "active" or "repressed" based on the local chromatin state (see Methods and Additional file 1: Tables S3 and S4). Genes were rank-ordered by expression level, binned (n = 500), and the fraction of genes in the bin with either active or repressed promoters was plotted against the genes’ mean expression level (Figure 1). As expected, ~100% of highly expressed genes have active promoters. However, transcripts detected at low levels tend to be associated with repressed promoters, suggesting that they do not play a functional role in the cell.

We judged that a reasonable expression cutoff describing the active genes in a cell would be the point where the ratio of active to repressed promoters drops below 1. Identifying this point by linear interpolation yielded FPKM values from 0.14 to 0.44 or log_2_(FPKM) values from −2.8 to −1.2 across the 9 ENCODE samples, a three-fold range of expression (Table 1). However, some of the variability in these values is explained by small positional shifts in the log_2_(FPKM) distributions. To normalize the distributions, we fit the right half of each gene expression curve to a half-Gaussian curve, mirrored the half-Gaussian into a full Gaussian distribution, and transformed log_2_(FPKM) into zFPKM derived from this fit (see Methods and Additional file 1: Table S2). After applying this transformation, and removing an outlier, we find that the active/repressed promoter threshold is zFPKM −2.82 +/− 0.22 (Table 1). Thus the zFPKM transform can be used with gene expression data alone to determine with high consistency the range of gene expression defined by active chromatin. Hereafter, we define this threshold as zFPKM > = −3, preferring to err on the side of capturing too many noisy genes rather than too few active ones.

**Table 1:** A Gaussian fit describes active genes

**Cell line**	**μ**	**σ**	**Threshold**	**Threshold**
			**log**_**2 **_**(FPKM)**	**zFPKM**
GM12878	3.70	1.94	−2.18	−3.03
H1-eSC	3.42	2.18	−1.20*	−2.12*
HMEC	3.77	2.11	−2.37	−2.91
HSMM	3.77	2.05	−2.41	−3.02
HUVEC	3.54	2.27	−1.85	−2.38
HepG2	3.24	2.18	−2.79	−2.77
K562	3.83	1.98	−2.19	−3.04
NHEK	3.45	2.07	−1.96	−2.61
NHLF	3.69	2.07	−2.06	−2.78
Mean +/− SD			−2.11 +/− 0.42	−2.74 +/− 0.30
			−2.23 +/− 0.28*	−2.82 +/− 0.22*

The distribution of log_2_ (FPKM) expression for each sample was calculated and the right side of the major peak was fit by a Gaussian distribution with parameters μ and σ. The threshold of active gene expression, defined as the intersection between the linear fit of the active promoter fraction and the repressed promoter fraction, was calculated in log_2_(FPKM) and zFPKM. (*) H1 embryonic stem cells were removed as an outlier.

**Figure 2:**
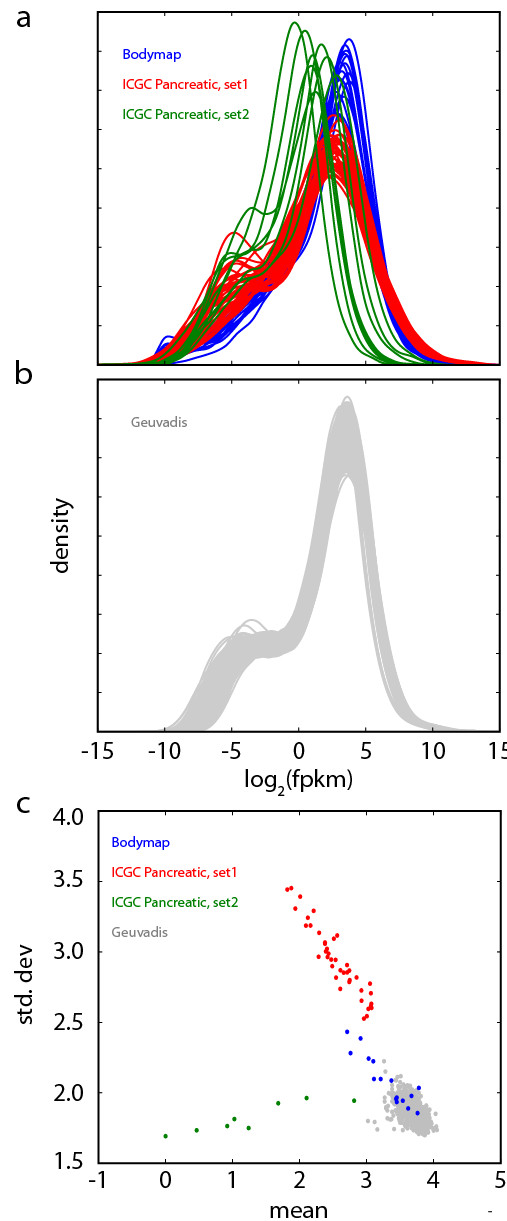
**RNA-seq data from different experimental protocols shows varied distributions of gene expression. (a)** Log_2_(FPKM) distributions from the Illumina Bodymap samples, ICGC Pancreatic cancer samples, and **(b)** the GEUVADIS RNA-seq data from 465 lymphoblastoid cell lines **(c)** A scatter plot of mean vs. standard deviation of the Gaussian fit for each of these experiments (bottom panel) shows the relative variation in the gene expression distributions.

Data from the ENCODE cell lines is the product of a controlled set of experimental and analytical protocols. It is therefore not surprising that the FPKM distributions are highly consistent; in fact, the normalized zFPKM threshold of −3 corresponds to a raw FPKM in a fairly tight range of 0.10 to 0.31 across the 17 ENCODE cell lines. However, many if not most other data sets lack this internal consistency. Figure 2 shows the log_2_(FPKM) distributions from several public data sets, including the Illumina BodyMap set of 16 healthy human tissues, pancreatic cancer RNA-seq from ICGC [ref], and the recently published GEUVADIS project RNA-seq of 465 lymphoblastoid cell lines derived from different individuals [15]. We fit a Gaussian to the major peak of each distribution and plotted the mean and standard deviation of each fit (Figure 2c). The resulting scatter plot demonstrates the variability of some RNA-seq data (and, conversely, the remarkable consistency of the GEUVADIS data), and strongly signals that a single heuristic for such diverse data may not be appropriate. The zFPKM approach offers a useful data normalization strategy in these cases.

While we do not have corresponding information on chromatin state for these samples, other cell line data do corroborate the relationship between promoter activation level and gene expression in the major peak. Additional file 2: Figure S2 shows RNA-seq distributions and corresponding paired histone H3K4 trimethylation ChIP-seq data. As with the Encode chromatin state data, the fraction of genes with promoter-associated H3K4me3 is high for genes expressed in the primary peak and drops to negligible levels for transcripts detected at trace levels.

#### The zFPKM threshold is robust to changes in read depth

**Figure 3:**
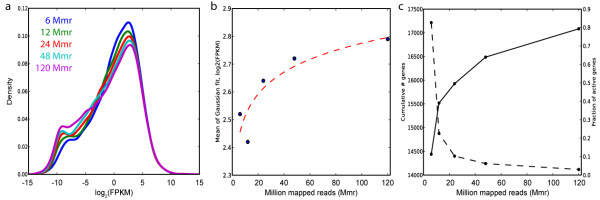
**The set of active genes is robust to read depth changes. (a)** RNA-seq of CD4+ memory T cells at different read depths. Deeper sequencing increases the fraction of total mass in the low-expression region of the log_2_(FPKM) distribution. **(b)** The Gaussian fit captures the read depth-induced variation in expression distributions. For gene expression distributions at each read depth (x-axis), the Gaussian fit was calculated and the mean plotted (y-axis) vs. read depth. Dashed line, linear regression of log(read depth) vs. mean of Gaussian fit. **(c)** As read depth increases (x-axis), the cumulative number of detected genes increases (solid line, left axis). However, the fraction of newly detected genes which are expressed in the active region drops (dashed line, right axis). Beyond 24 Mmr, only 58 new active genes are detected.

To evaluate the robustness of the zFPKM transform, we applied it to RNA-seq data derived from different read depths. Human CD4+ memory T cells were costimulated with anti-CD3/CD28 beads for 48 hours and RNA-seq was performed using the Illumina platform, yielding a total depth of ~120 million mapped reads (Mmr). Subsets of reads, with depths at 6, 12, 24, 48, and 120 Mmr, were analyzed using the same pipeline. Increasing read depth has two main effects on the log_2_(FPKM) distribution: it increases the proportion of mass in the noisy left shoulder (Figure 3a), and it subtly shifts the main peak of the distribution (Figure 3b). This occurs because, as deeper sequencing discovers new transcripts (Figure 3c), each doubling of mapped reads is divided across a larger number of genes, thus subtly lowering the inferred FPKM of moderate-expression genes (which counterintuitively right-shifts the curve fit). The zFPKM transform normalizes this shift and captures essentially the same set of active genes (12,475 +/− 176) across all read depths, with a coefficient of variation of less than 1.5%.

As noted in this study and others [5], greater read depth increases the total number of genes detected (Figure 3c, solid curve), with newly discovered genes tending to show very low expression (Additional file 2: Figure S3). The corresponding fraction of newly detected genes that are expressed in the active region drops rapidly with read depth (Figure 3c, dashed curve). Beyond 24 Mmr, though transcripts from over 1,000 new genes are putatively detected, only 37 are observed in the active region. This suggests 20–30 Mmr is a reasonable target for RNA-seq studies of gene expression, as it captures virtually all active genes in a sample while allowing sample multiplexing on sequencing machines to reduce costs. This result is consistent with ENCODE recommendations for RNA-seq best practices [16]; moreover, at an expression level of log_2_(FPKM) of −2.8 (the lowest expression level corresponding to our zFPKM threshold in the samples studied here), this read depth yields ~10 mapped reads per typical 3 kb transcript, the minimum coverage recommended for analysis of differential expression using count-based statistics [17].

Other normalization methods have been proposed to deal with the change in calculated FPKM induced by, e.g., changes in read depth and mapping quality. One such option is transcripts per million (TPM), implemented in the RSEM software package [18] and used to compute gene expression values in, e.g., The Cancer Genome Atlas [19]. While the TPM transform should in principle be more stable than raw FPKM, the software implementation (rsem-calculate-expression version 1.2.6 at time of writing) calls Bowtie with lax mapping parameters that result in dozens to hundreds of genes being called highly expressed in one pipeline vs. trace or zero expression in the other. Additional file 2: Figure S4 shows the Tophat/Cufflinks-derived FPKM vs. RSEM-derived TPM for nine ENCODE cell lines and highlights the genes unique to each pipeline. Comparing the fraction of active and repressed promoters among these genes suggests that the default Tophat/Cufflinks pipeline delivers more accurate results (Additional file 2: Table S5), and that end-users should carefully consider the command line parameters when using RSEM as a wrapper for Bowtie.

## Conclusions

Second-generation sequencing technology has provided a detailed view of the transcriptome. Assays that previously required multiple platforms, or which were simply not available, now can be performed from a single sequencing data set; e.g. transcript quantitation, isoform identification, alternative splicing and transcription start sites, allele-specific transcription, and discovery of novel transcripts. For common assays of gene expression, however, the remarkable sensitivity of RNA-seq has generated many questions regarding how to most efficiently design an experiment and analyze the resulting data.

Previous transcriptome studies have suggested that many rare transcripts may be the product of biological noise, although few have provided evidence that these products are non-functional. We show that low-abundance transcripts are associated with chromatin signatures consistent with repressed promoters, and we provide the zFPKM normalization method that accurately determines the expression regime defined by genes controlled by active promoters. The method provides several advantages over widely used heuristic approaches of accepting expression values above a fixed threshold, typically FPKM values ~ 1. We show that, while most human RNA-seq experiments yield similarly shaped distributions of gene expression values, different samples and experimental protocols can result in pronounced changes in the location and scale of these distributions that add variability to the results from the application of such heuristics. In the more extreme cases, however, it would be worth carefully re-evaluating the quality of the primary data before applying any normalization techniques.

With a finite population of biologically active transcripts in a cell, it stands to reason that experimental methods can be optimized to provide requisite coverage of those transcripts while maximizing the multiplexing capability of a sequencer. Our work shows that 20–30 million mapped reads are sufficient to detect virtually all active transcripts in a cell line, and provides deep enough coverage to undertake analysis of differential expression across the bulk of the active transcriptome. RNA-seq at ever greater depth continues to detect new transcripts, but the overwhelming majority are expressed at trace levels and, in the ENCODE data, are associated with repressed promoters, indicating that these are not biologically active genes.

It is worth noting that these results are derived primarily from homogeneous samples of human cell lines. Heterogeneous samples present their own set of challenges. A gene that is moderately expressed in a small fraction of cells in a sample might be indistinguishable from the background transcripts of the whole sample. At the other extreme, an equal mix of two or three cell types would likely result in a similar top-end distribution of constitutively expressed genes but an enlarged left shoulder of tissue-specific genes whose observed expression is reduced by averaging over the whole sample. While none of these issues are unique to RNA-seq—microarray studies have long faced the same problems—there may be an opportunity to formally quantify this behavior by *in silico* combinations, across a range of proportions, of the ENCODE matched transcript and chromatin state data from different samples.

More broadly, the ENCODE data provides a unique and comprehensive data set from which to evaluate the quality of RNA-seq studies generally. Having independent chromatin state data for multiple cell lines provides a vital "ground truth" against which to measure the performance of RNA-seq analysis tools. We point to the differences between RSEM and Tophat/Cufflinks quantitation presented here as a case study for using this framework to evaluate computational methods against real-world data.

Sequencing technology has evolved significantly since the early proof-of-concept RNA-seq studies. Through a combination of bioinformatic and biochemical advances, modern RNA-seq data represents a deeper and more accurate sampling of the transcriptome than the moderate-depth, short-read data from which many current rules of thumb for analysis were derived. Improved library prep techniques have increased the fraction of total sequenced bases that map to mRNA and reduced the bias toward reads mapping at the 3′ end of known transcripts, while splice-aware mappers align longer reads with greater accuracy and less likelihood of multiple hits. The net result is that many of the features of early RNA-seq data which drove the development of heuristics in use today are not always applicable. We evaluate latest-generation data and offer an updated framework for extracting relevant gene expression information from RNA-seq experiments.

## Methods

### Data sources

ENCODE RNA-seq data were downloaded from NCBI GEO (Accession no. GSE30567). Jurkat RNA-seq and ChIP-seq and CD4+ RNA-seq data generated in the Salomon lab were submitted to GEO. From EMBL-EBI, we downloaded Illumina BodyMap reads [E-MTAB-513] and Geuvadis FPKM values [E-GEUV-1]. Other sequence data were acquired from NCBI SRA: HeLa RNA-seq, SRR309265; HeLa ChIP-seq, SRR037862; HCC1954 RNA-seq and ChIP-seq, SRX061987-SRX061997. ICGC pancreatic cancer RNA-seq FPKM values were downloaded from ftp://data.dcc.icgc.org/current/Pancreatic_Cancer-OICR-CA/.

### Cell culture

For isolation of total CD4 T cells and memory CD4 T cells, peripheral blood mononuclear cells (PBMCs) were first enriched by density gradient centrifugation of peripheral blood from healthy human donors through a Ficoll-histopaque gradient (Sigma). For total CD4 T cell purification, cells were positively selected from PBMCs on anti-CD2 beads (Miltenyi) followed by positive selection on anti-CD4 beads (Invitrogen). CD4+ memory T cells were purified from PBMCs by negative selection with magnetic beads (Miltenyi). Purified cells were cultured in RPMI (Mediatech) supplemented with 10% FBS, 100 U/ml Penicillin (Gibco), and 100 μg/ml Streptomycin (Gibco) for 48 hours with and without stimulation by anti-CD3/CD28 beads (Invitrogen) at 37C in 5% CO2. Jurkat cells were obtained from ATCC (clone E6-1) and cultured in the same medium as the primary cells.

### RNA-seq

Cells were harvested and resuspended in TRIzol (Invitrogen). RNA was isolated following a standard TRIzol extraction protocol. RNA-seq libraries were prepared as described [20]. Briefly, 100 ng total RNA was amplified using the Ovation RNA-seq kit (NuGen). 100 ng amplified cDNA was digested with 50 U/µl endonuclease S1 (Promega) for 30 min at room temperature. Digested cDNA was end repaired and sequencing adapters were annealed following standard protocols (Illumina). Sequencing of total CD4 T cell RNA was conducted on an Illumina GAIIx instrument with 60-base paired-end reads. Ultradeep sequencing of activated CD4 memory T cell RNA was conducted in 5 lanes of the Genome Analyzer IIx instrument, generating 80-base single-end reads.

### Sequence mapping and gene expression quantitation

RNA-seq reads were mapped to hg19 with TopHat version 1.4.1. No junctions file (−j) or GTF file (−G) was specified. FPKM values were calculated per gene with Cufflinks version 2.0.2, using the Gencode v.14 GTF file downloaded from the Human Genome Browser at UCSC. Cufflinks output was filtered for protein coding genes as annotated by the HUGO Gene Nomenclature Committee (http://www.genenames.org). The matrix of raw FPKM values is included as Additional file 1: Table S1.

### Gaussian fit and zFPKM normalization

For protein-coding gene expression values for each cell line, log_2_(FPKM) values less than −15 were set to not-detected. An empirical distribution of log_2_(FPKM) values was calculated by kernel density estimate in Python using scipy.stats.gaussian_kde with default parameters. A half-Gaussian curve was fitted to the right half of the main peak by setting *μ* at the kde maximum. The standard deviation is then determined by:

$$\sigma =\left(U-\mu \right)\sqrt{\frac{\pi }{2}}$$

where *U* is the mean of all log_2_(FPKM) values > *μ* The half-Gaussian was then mirrored to a full Gaussian distribution with parameters (*μ*, *σ*). Log_2_(FPKM) is then transformed to zFPKM:

$$\mathrm{zFPKM}=\frac{\log_{2}\left(\mathrm{FPKM}\right)-\mu }{\sigma }$$

A matrix of all calculated zFPKM values is included as Additional file 1: Table S2.

### Promoter chromatin state

Files containing the results of chromatin state analysis in [14] were downloaded in .bed format from the Human Genome Browser at UCSC at http://hgdownload.cse.ucsc.edu/goldenpath/hg19/encodeDCC/wgEncodeBroadHmm/. In a given cell line, a gene was labeled as having an active promoter if a locus was classified as State 1 ("Active Promoter") or State 2 ("Weak Promoter") within 2 kb of the transcription start site of any annotated transcript associated with the gene in the Gencode gene models. A gene was labeled as having a repressed promoter if any TSS was within a locus classified as State 12 ("Polycomb-repressed") or State 13 ("Heterochromatin"). In rare cases genes were labeled with both active and repressed promoters. A list of all active and repressed genes in each sample is included as Additional file 1: Tables S3 and S4.

### ChIP-seq

ChIP-seq reads were mapped to hg19 with Bowtie, and peak finding was performed using sissrs [21]. H3K4me3 peaks within 1 kb of a gene transcription start site were identified based on the GTF file described above.

### Ethics statement

All data generated in the Salomon lab for this manuscript were covered by Human Subjects Research Protocols approved by the Institutional Review Board. Informed written consent was obtained from all study subjects in the study.

## Competing interests

The authors declare that they have no competing interests.

## Authors’ contributions

TH designed the study and carried out the analyses, and drafted the manuscript in collaboration with DS. HKK, SL, and KP performed RNA-seq and ChIP-seq experiments, analyzed resulting data, and collaborated in analytical design. All authors read and approved the final manuscript.

## Supplementary Material

Additional file 1: Tables S1-S4 containing FPKM, zFPKM, and promoter classification for each gene. Click here for file

Additional file 2: Figure S1 Encode cell line log2(FPKM) distributions (blue), Gaussian fits to the major peak (red), fraction of binned genes with active promoters (green), and fraction of binned genes with repressed promoters (black). **Figure S2.** Cell line log2(FPKM) distributions (blue), mirrored half-Gaussian fits to the right side of the major peak (red), and fraction of binned genes (n=500) with H3K4me3 within 1kb of a promoter (green; right axis). **Figure S3.** With increasing read depth (x-axis), RNA-seq of CD3/CD28 costimulated memory CD4+ cells detects an increasing number of transcripts (red; left axis). **Figure S4.** Tophat/Cufflinks vs RSEM quantitation. **Table S1.** Tophat/Cufflinks vs. RSEM quantitation. Click here for file
