# Supplementary material for: Finding the active genes in deep RNA-seq gene expression studies
Source: BMC Genomics. 2013 Nov 11;14:778. doi: 10.1186/1471-2164-14-778 (PMC3870982; doi:10.1186/1471-2164-14-778)
Supplement: Additional file 2: Figure S1 — Encode cell line log2(FPKM) distributions (blue), Gaussian fits to the major peak (red), fraction of binned genes with active promoters (green), and fraction of binned genes with repressed promoters (black). Figure S2. Cell line log2(FPKM) distributions (blue), mirrored half-Gaussian fits to the right side of the major peak (red), and fraction of binned genes (n=500) with H3K4me3 within 1kb of a promoter (green; right axis). Figure S3. With increasing read depth (x-axis), RNA-seq of CD3/CD28 costimulated memory CD4+ cells detects an increasing number of transcripts (red; left axis). Figure S4. Tophat/Cufflinks vs RSEM quantitation. Table S1. Tophat/Cufflinks vs. RSEM quantitation. [file 1471-2164-14-778-S2.docx]

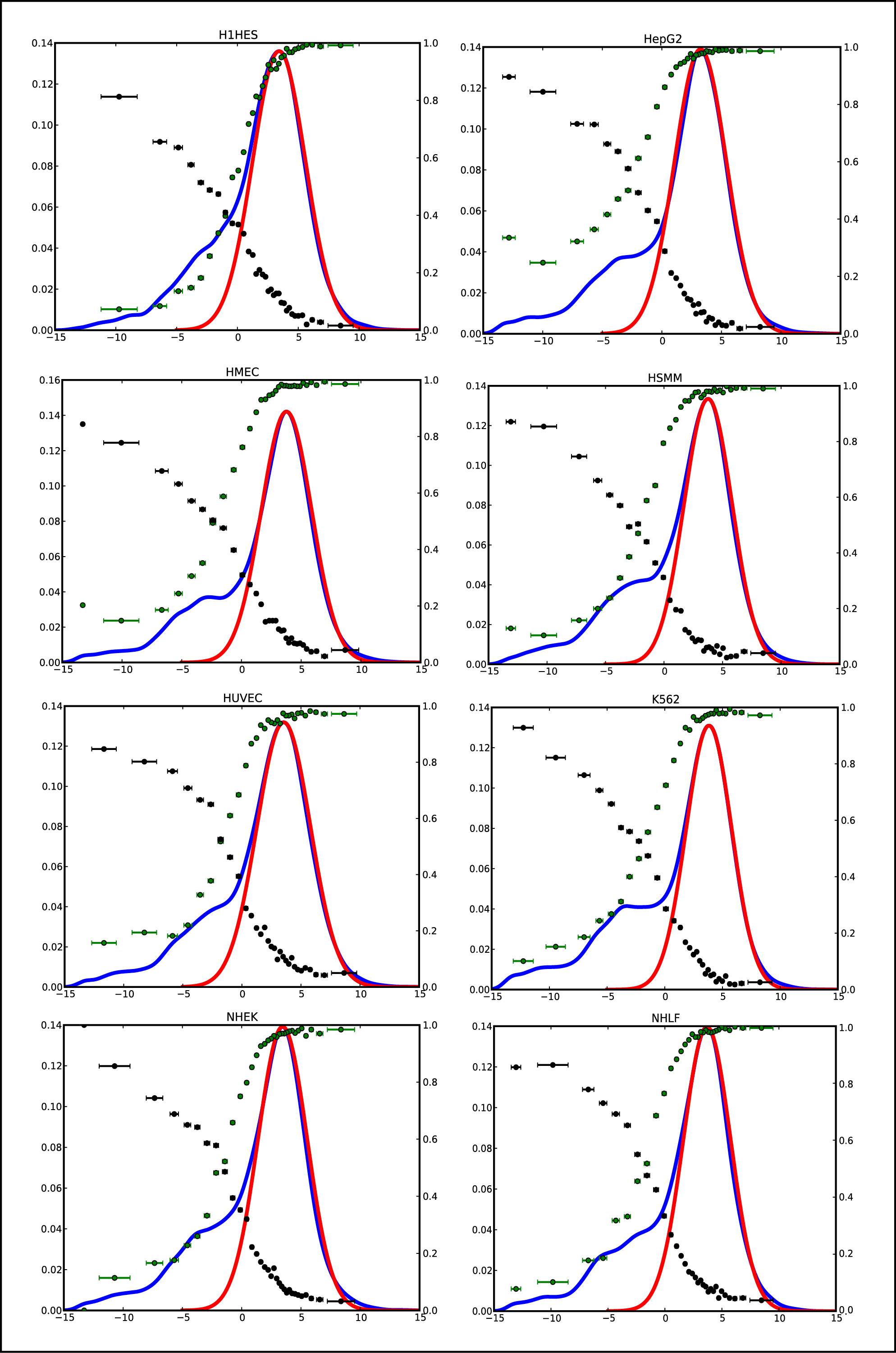


Figure S1. Encode cell line log2(FPKM) distributions (blue), Gaussian fits to the major peak (red), fraction of binned genes with active promoters (green), and fraction of binned genes with repressed promoters (black).


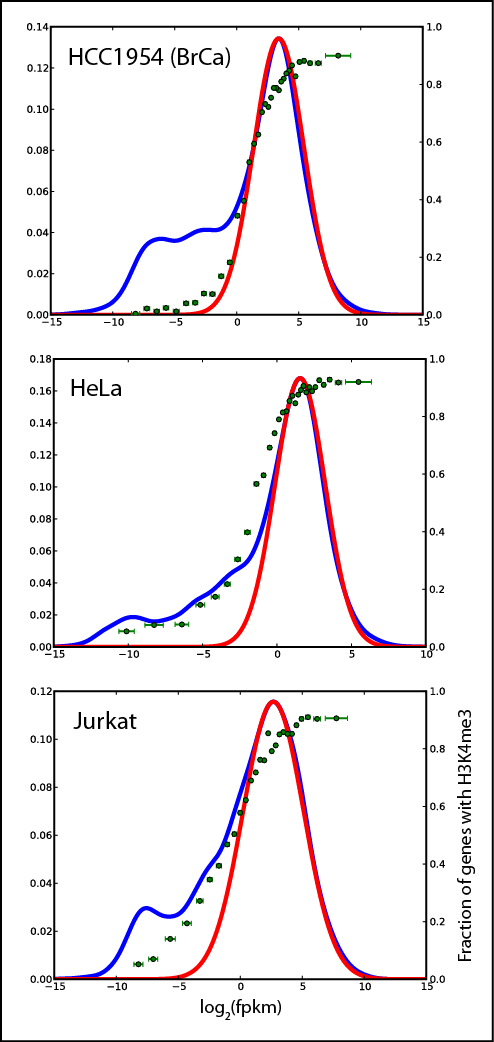


Figure S2. Cell line log2(FPKM) distributions (blue), mirrored half-Gaussian fits to the right side of the major peak (red), and fraction of binned genes (n=500) with H3K4me3 within 1kb of a promoter (green; right axis).


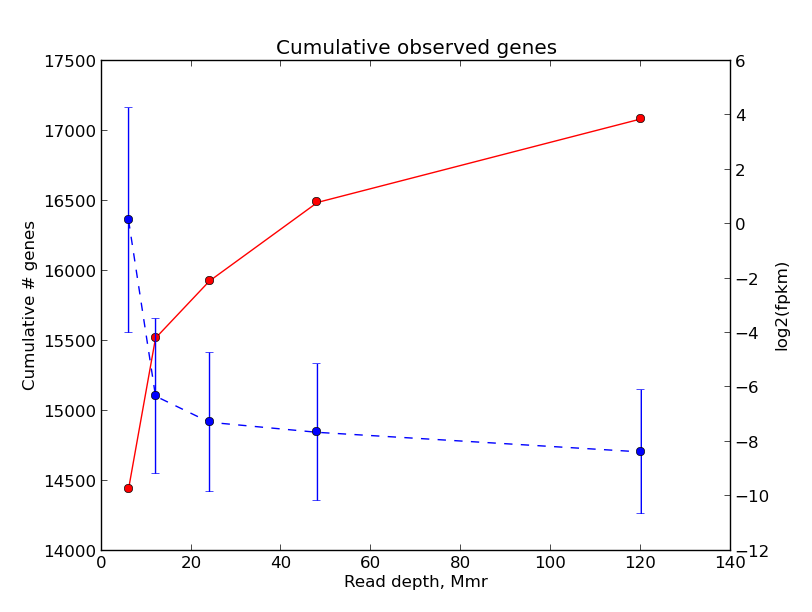


Figure S3. With increasing read depth (x-axis), RNA-seq of CD3/CD28 costimulated memory CD4+ cells detects an increasing number of transcripts (red; left axis). At each depth, newly discovered genes show lower gene expression (blue, mean +/- s.d., right axis)


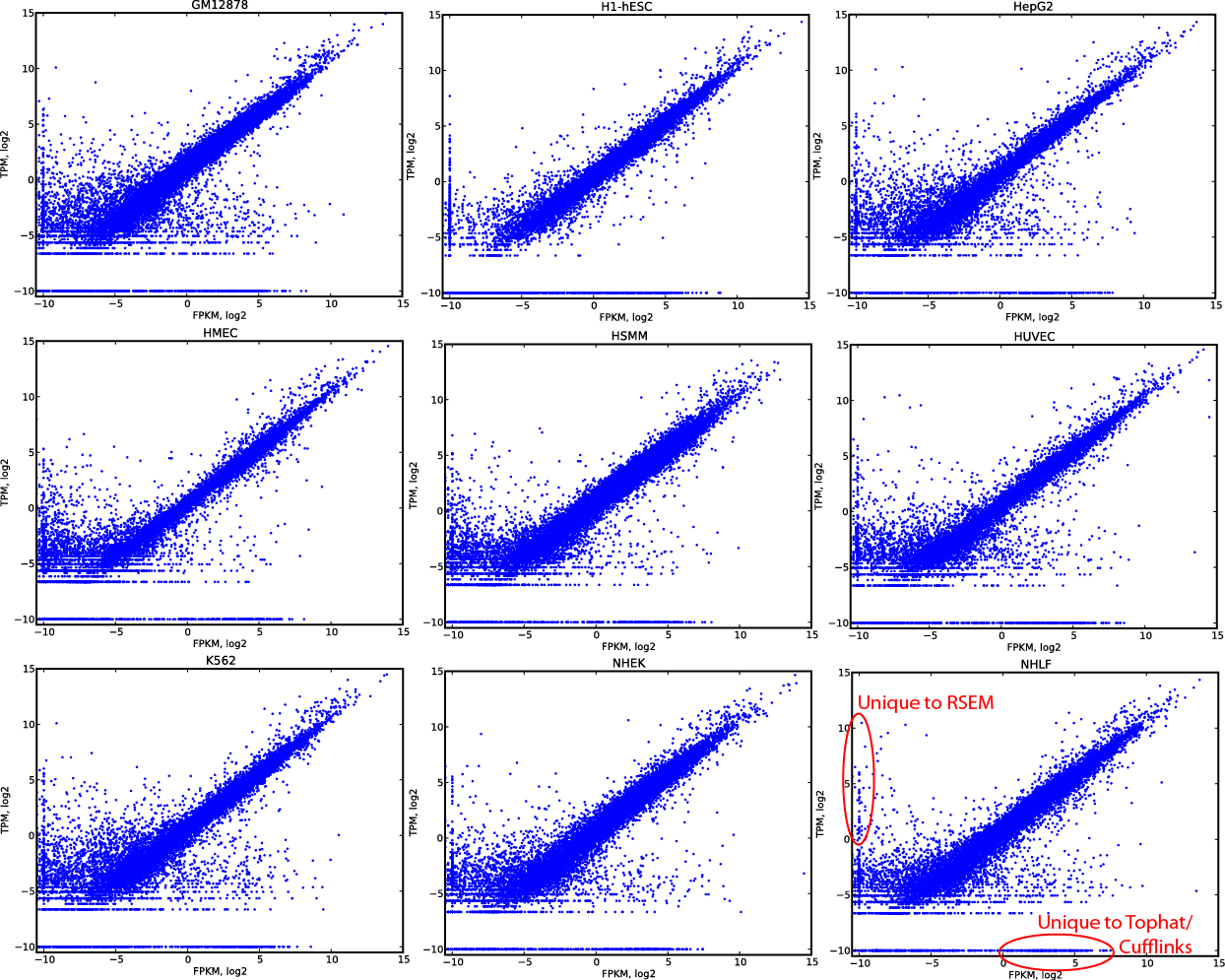


Figure S4. Tophat/Cufflinks vs RSEM quantitation. RNA-seq data for Encode cell lines was analyzed with default Tophat/Cufflinks (x-axis) and RSEM (y-axis) pipelines. While quantitation is generally highly correlated, each pipeline uniquely discovers dozens to hundreds of genes (see Supplementary Table 5)

**Supplementary Table 5**: Tophat/Cufflinks vs. RSEM quantitation. Each pipeline, by default, calls bowtie with substantially different parameters. While resulting gene quantitation is broadly consistent (See Fig S4), dozens to hundreds of genes are uniquely assigned moderate to high expression values (log_2_(fpkm or tpm) > 0) in one pipeline but are absent in the other (log_2_(fpkm,tpm) < -5). Genes unique to the Tophat/Cufflinks pipeline show active chromatin signatures; those unique to RSEM show repressed chromatin signatures.

|  | **Tophat/Cufflinks:FPKM** | | | **RSEM:TPM** | | |
| --- | --- | --- | --- | --- | --- | --- |
|  | **Genes** | **active** | **repressed** | **Genes** | **active** | **repressed** |
| GM12878 | 241 | 64.7% | 34.4% | 204 | 20.1% | 75.0% |
| H1-hESC | 189 | 89.4% | 15.3% | 37 | 54.1% | 40.5% |
| HMEC | 195 | 84.6% | 19.0% | 162 | 14.8% | 74.7% |
| HSMM | 202 | 84.7% | 19.3% | 124 | 21.0% | 74.2% |
| HUVEC | 220 | 72.7% | 35.0% | 166 | 17.5% | 77.7% |
| HepG2 | 237 | 76.0% | 27.0% | 205 | 30.7% | 79.0% |
| K562 | 243 | 67.5% | 29.6% | 257 | 16.3% | 80.2% |
| NHEK | 222 | 75.7% | 29.3% | 108 | 20.4% | 77.8% |
| NHLF | 236 | 72.0% | 30.9% | 142 | 21.1% | 76.8% |
| *mean* | **220.56** | **76.4%** | **26.7%** | **156.11** | **24.0%** | **72.9%** |
